# A Systematic Review and Meta-Analysis of Eltrombopag Efficacy Combined With Immunosuppressive Drugs in Treatment of Severe Aplastic Anemia

**DOI:** 10.7759/cureus.65970

**Published:** 2024-08-01

**Authors:** Janan Illango, Kofi D Seffah, Namballa Naveen, Yubraj Paudel, Anandkumar Patel, Vamsi krishna Pachchipulusu, Heet N Desai, Beenish Najam, Pousette Hamid

**Affiliations:** 1 Research, California Institute of Behavioral Neurosciences and Psychology, Fairfield, USA; 2 Internal Medicine, Phoebe Putney Memorial Hospital, Albany, USA; 3 Internal Medicine, California Institute of Behavioral Neurosciences and Psychology, Fairfield, USA; 4 Internal Medicine, Piedmont Athens Regional Medical, Athens, USA; 5 Internal Medicine, Steel Authority of India Limited (SAIL) Hospital, Dhanbad, IND; 6 Internal Medicine, Pushpanjali Hospital Pvt. Ltd., Bharatpur, NPL; 7 Neurology, California Institute of Behavioral Neurosciences and Psychology, Fairfield, USA; 8 Neurology, Shalby Hospital Naroda, Ahmedabad, IND; 9 Medicine, Maharshi Hospital Private Limited, Surendranagar, IND

**Keywords:** thrombopoietin receptor agonist, hematopoietic stem cell transplantation, immunosuppressive therapy, aplastic anemia, eltrombopag

## Abstract

Severe aplastic anemia (SAA) is a life-threatening disorder with high mortality. The only curative treatment is hematopoietic stem cell transplantation (HSCT), but it is mainly for young patients with suitable donors. The alternative is immunosuppressive therapy (IST), which can improve blood counts in about 58% of patients, but many relapse after discontinuation. Recently, eltrombopag, a thrombopoietic receptor agonist, was tested. As a single drug, it improved blood counts in 40-50% of patients. However, combining eltrombopag and IST proved more effective and safer. A review of 20 randomized controlled trials with 2,469 patients showed that the group receiving eltrombopag and IST had a significantly higher overall response rate (86% vs. 74%) after six months. After two years, 54% of the experimental group had relapsed compared to 39% in the control group. Despite this, eltrombopag tends to increase relapse rates over time. In conclusion, combining eltrombopag with IST is a superior treatment for SAA.

## Introduction and background

Aplastic anemia (AA) is a rare hematological disorder caused by impaired bone marrow function [1]. Its incidence ranges from two to three cases per million per year in Europe and North America and five to six cases per year in Asia. The disease is more prevalent in younger populations between the ages of 15 and 25 years and people older than 60, but no differences have been identified between genders or ethnic groups [2]. The extent of bone marrow impairment directly correlates with the clinical presentation of the disease. In most cases, bone marrow appears hypoplastic and produces a low number count of all hematopoietic stem cells (HSCs). Clinically, this presents with signs and symptoms related to anemia, leukopenia, and thrombocytopenia [1]. In rare cases, bone marrow aplasia may occur and lead to the clinical presentation of severe pancytopenia, where the production of all HSCs is severely compromised [3]. However, the etiology of AA remains unclear [2]. An autoimmune reaction can also be initiated by these pathogenic factors, which stimulate the cytotoxic activity of T cells and the production of cytokines. Eventually, this immune response destroys HSCs and progenitor cells, leading to bone marrow failure. Inherited bone marrow failure syndromes (IBMFS), such as Fanconi anemia (FA), Dyskeratosis congenita (DC), and Schwachman-Diamond syndrome (SDS), may progress into inherited AA. Genetic disorders that cause IBMFS are mutations in different bone marrow genes [1,2].

The diagnosis of AA can be established by examining peripheral blood counts and bone marrow morphology. In the early stages of the disease, the peripheral blood morphology demonstrates different cytopenias (mono or bipenia) with erythrocytes macrocytosis and lymphocyte count within normal range limits. In late stages, pancytopenia with varying severity may be observed. Based on the hematological values of blood cells and bone marrow cellularity, AA can be classified into moderate, severe, and very severe AA (VSAA). Diagnostic criteria for SAA are as follows: (1) bone marrow cellularity <25% and (2) severe pancytopenia (neutrophils <0.5 × 10^9^ /L, platelets <20 × 10^9^ /L and reticulocytes <20 × 10^9^ /L) [1,4-5].

Severe aplastic anemia (SAA) is a life-threatening condition with an inferior prognosis if left untreated. In cases of VSAA, the mortality rate can be as high as 90% [5]. The current standard-of-care treatments for SAA are hematopoietic stem cell transplantation (HSCT) and immunosuppressive therapy (IST) [1-2,4-5]. HSCT is typically recommended for individuals under 40 if fully matched human leukocyte antigen (HLA) donors are available. In all other cases, the only available treatment option is IST, as various auto-immune mechanisms are involved in presenting acquired AA [4]. Anti-thymocyte globulin (ATG) and cyclosporine (CSA) are two drugs that have shown the highest hematologic response rate (55 %) in SAA patients [5]. ATGs are available in different types, which depend on the animal used for their production. The ATG derived from rabbits, horses, or pigs is most commonly used in the treatment of AAA [6].

Previously published studies have shown that IST in children with SAA had a high overall response (OR = 70%), complete response rate (CR = 23-60%), and overall survival (OS = 80-93%). However, the long-term event-free survival (EFS) rates were low (56-62%) because IST was associated with long-term complications such as relapse and clonal evolution11 [4]. To improve response rates, lymphotoxic agents, such as cyclophosphamide, alemtuzumab, and mycophenolate, were combined with IST. However, the results were unsatisfactory [7]. In other studies, different growth factors, such as erythropoietin, granulocyte colony-stimulating factor (G-CSF), stem cell factor, and interleukins, were added to IST to increase the production of blood cells and improve response rates. However, the results did not show any significant difference in response rates among patients who received a combination of growth factors and IST compared to IST alone [8]. Thrombopoietic (TPO) receptor agonists, including eltrombopag (EPAG), have shown promise in treating SAA. TPO is a known treatment option for immune thrombocytopenia (ITP), but its effects and mechanisms of action in SAA patients are unclear [9, 10]. Recent in vitro studies have discovered that EPAG, by binging to the c-Mpl receptor, may stimulate the proliferation of all HSCs and not only megakaryocytes [11-14]. The efficacy of EPAG was first studied in patients with refractory SAA (rSAA) as a single therapy at doses of 150 mg/day. The overall response rate (ORR) was about 40-50%, but when combined with horse ATG and CSA, the response rates were up to 94% [15,16]. In February 2021, the United States Food and Drug Administration (FDA) approved EPAG, combined with IST, for the treatment of SAA in children aged two and above [4]. A recent study has emphasized the importance of supportive treatment for SAA patients. Although there have been significant improvements in the treatment of SAA in the last decade, it has been confirmed that certain measures, such as transfusions with leukocyte-depleted blood products, anti-infectious prophylaxis, and treatment of severe infections with intravenous broad-spectrum antibiotics combined with antifungal, antiviral agents and G-CSF, can significantly improve the overall survival rate from SAA, especially in elderly patients with comorbidities [2].

This review paper aims to evaluate the effectiveness of EPAG in the treatment of SAA patients. To achieve this, we conducted a meta-analysis and compared the overall response and relapse rates (RRs) of patients who received standard IST with those who received a combination of EPAG and IST.

## Review

Methods

Search Strategy

For this review, we searched through articles in one of the largest and most comprehensive databases of biomedical and life sciences literature. The PubMed database contains over 36 million citations and abstracts with good-quality data results. It provides a search for medical articles indexed with the medical subject heading (MeSH) terms and journals reviewed and selected by the National Library of Medicine through MEDLINE and PubMed Central database. To conduct our search, we used the following MeSH parameters: “aplastic,” “anemia,” “acquired,” and “treatment.” We started by searching for “aplastic anemia” and found 21,502 journals. Then, we narrowed our search to “acquired aplastic anemia” and found 1,607 journals. Finally, we filtered our search with the MeSH criteria, “acquired aplastic anemia treatment,” and found 988 relevant articles.

Selection Criteria

We used the PICO (Population/Intervention/Comparison/Outcome) to define study inclusion criteria and formulate valid research questions as presented in Table 1.

**Table 1:** PICOT table PICOT - Population/Intervention/Comparison/Outcome/Time

Question	What is the effect of eltrombopag combined with immunosuppressive drugs in severe aplastic anemia patients?
P - Patient, population, or problem	Children older than two years and adult patients diagnosed with SAA
I - Intervention, exposure, prognostic factor	Eltrombopag combined with immunosuppressive drugs
C - Comparison	Immunosuppressive drugs
O - Outcome	Overall response rate and relapse rate
T - Time factor, type of study (optional)	Double-blind and randomized controlled trials, systemic reviews, and meta-analysis

A total of 988 articles were initially selected, but we further filtered them using the following inclusion criteria. First, the articles must be available for free and in full text. Second, they had to be clinical trials, meta-analyses, randomized controlled trials (RCTs), or systematic reviews. Third, they had to be written in English. Fourth, the studies had to be conducted on human participants. Fifth, they had to be published within the last year (2021). We applied several filters to a set of articles and ended up with 433 full-text articles that were free to access. Out of these, 123 articles met our criteria for clinical trials, meta-analyses, reviews, and systematic reviews. Only 122 of these articles were published in English. Among the 123 selected articles, 121 of them contained clinical trials conducted on humans. Our population included children older than two years and adult patients diagnosed with SAA.

To refine our search, we utilized specific MeSH terms such as “immunosuppressive agents,” “cyclosporine, therapeutic use,” “hematopoietic stem cell transplantation,” and “thrombopoietin-receptor agonist.” This helped us narrow down our results to only three articles that met our inclusion criteria for SAA as a combination of EPAG and IST or IST alone. Four other articles were excluded from our search as they were irrelevant to treating SAA but focused on bone marrow transplantation outcomes and supportive care. Based on inclusion criteria for ORR and RR, only nine RCTs were included in our meta-analysis.

**Figure 1:**
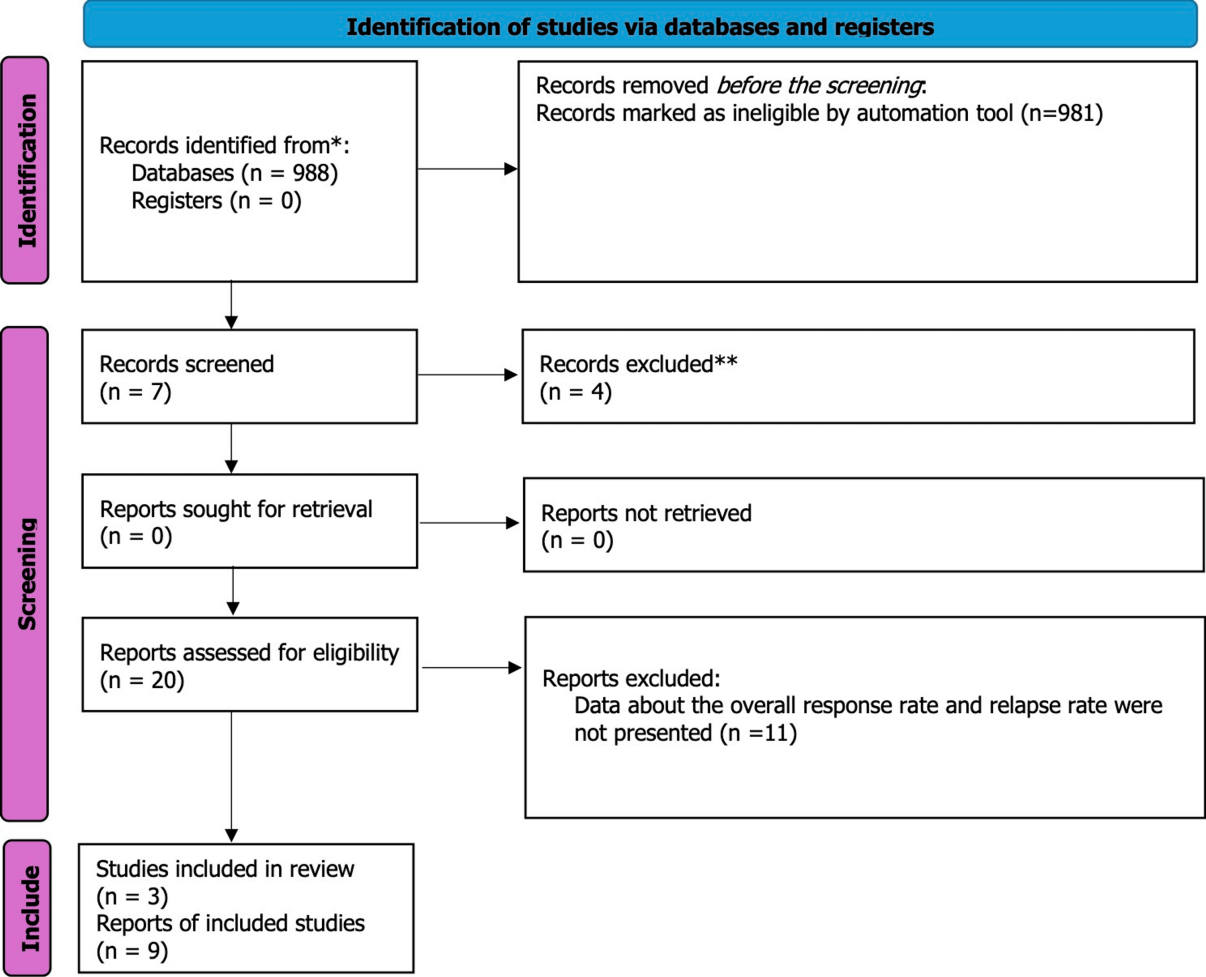
Preferred Reporting Items for Systematic reviews and Meta-Analyses (PRISMA) diagram 2020

We utilized the Preferred Reporting Items for Systematic Reviews and Meta-Analyses (PRISMA) diagram of 2020 to present our systematic review of the PubMed database search [17] (Figure 1).

Figure 1 shows that only three articles met the requirements for this review, which were studies on the treatment of SAA with EPAG and IST. Each article included data from RCTs conducted on SAA patients who received either IST or a combination of EPAG and IST. We only included RCTs that used the same parameters (hematologic response rate, complete response rate, relapse, clonal evolution, EFS, and overall survival rate) to assess treatment outcomes. This was done to enable comparison and validation of the study results. A hematologic response is considered when the levels of blood cells recover in one or more lineages. An increase in platelet count to 20 × 10^3^ /mL above the baseline or stable platelet counts with independence for transfusion for at least eight weeks in those who were transfusion-dependent is defined as a platelet response. The erythroid response is an increase in hemoglobin by 15g/L or a reduction in the units of packed red blood cells (RBC) transfusions of at least four transfusions for eight consecutive weeks in transfused patients. An increase in the absolute neutrophil count (ANC) to 0.5 × 10^3^ /mL is defined as a neutrophil response. A complete hematological response is when all three lineages recover, while the recovery of at least one lineage is defined as a partial response [1].

We conducted a meta-analysis of data from 20 different RCTs to evaluate the efficacy of EPAG in treating SAA patients when used in conjunction with standard IST. Our analysis compared the ORRs and RRs of patients treated with EPAG and standard IST to those treated with only standard IST. The data were gathered from three journals, each of which presented data from multiple RCTs. The list of RCTs included in our meta-analysis can be found in Table 2. We extracted data from each RCT and compiled it in a standardized Excel (Microsoft Corporation, Redmond, WA) table, including information about the first author, year of publication, patient characteristics, treatment regimen, EPAG dose, ORR, and RR. Statistical analysis was conducted on the collected data using XLSTAT software (v. 2021) (Lumivero, Denver, CO). We used various statistical tests to validate the difference between the two groups. We conducted the chi-squared test to examine whether there was any variation in the data collected from various RCTs. It is necessary to check for heterogeneity in the data before performing a meta-analysis to ensure that the outcomes are consistent and not due to chance. If p > 0.01, it means that heterogeneity is present. Whenever we found heterogeneity, we performed Cochran’s homogeneity test and I^2^ test. If the p-value of Cochran’s was less than 0.10, it indicated that the variability across RCTs was larger than what was expected by chance. The I^2^ test determines the percentage of variability in results across RCTs that were due to real differences. If the I^2^ test is less than 25%, heterogeneity is low. If it is between 25% and 50%, the heterogeneity is moderate. If it is over 50%, it shows high heterogeneity. To determine if there was an association between treatment and ORR or RR, we calculated the odds ratio (OR) with 95% confidence intervals (CI).

**Table 2:** General characteristics of patients on eltrombopag and immunosuppressive therapy ATG, anti-thymocyte globulin; CSA, cyclosporine; EPAG, eltrombopag; SAA, severe aplastic anemia; VSAA, very severe aplastic anemia

Selected journals	List of trials included in meta-analysis	Number of patients	Indication	Age (years)	Treatment	Eltrombopag dose
Dexler B, Passweg J: Current evidence and the emerging role of eltrombopag in severe aplastic anemia. (2021)	Townsley DM, et al. [15].	30	SAA/VSAA	>18	EPAG/ATG/CSA	150 mg
		31	SAA/VSAA	>18	EPAG/ATG/CSA	150 mg
		31	SAA/VSAA	>18	EPAG/ATG/CSA	150 mg
	Assi R, et al. [18]	21	SAA	>18	EPAG/ATG/CSA	150 mg
	Peffault de Latour R, et al. [19]	96	SAA/VSAA	>18	EPAG/ATG/CSA	150 mg
Zhang J, Wu Y, Liu J, et al.: A systematic review and meta-analysis of the safety and efficacy of anti-thymocyte globin combined with eltrombopag in the treatment of severe aplastic anemia. (2021)	Scheinberg P, et al. [6]	26	SAA	>18	EPAG/ATG	150 mg
	Bacigalupo A. [20]	48	SAA	>18	EPAG/ATG	150 mg
	Tichelli A, et al. [8]	36	SAA	>18	EPAG/ATG	150 mg
	Rogers ZR, et al. [21]	36	SAA	>18	EPAG/ATG	150 mg
	Hayakawa J, et al. [22]	26	SAA	>18	EPAG/ATG	150 mg
	Yang N, et al. [23]	38	SAA	>18	EPAG/ATG	150 mg
	Lengline E, et al. [24]	34	SAA	>18	EPAG/ATG	150 mg
	Sasaki N, et al. [25]	40	SAA	>18	EPAG/ATG	150 mg
	Li F, et al. [26]	42	SAA	>18	EPAG/ATG	150 mg
	Sharma R, et al. [27]	54	SAA	>18	EPAG/ATG	150 mg
	Bevans MF, et al. [28]	46	SAA	>18	EPAG/ATG	150 mg
	Frickhofen N, et al. [29]	32	SAA	>18	EPAG/ATG	150 mg
	Chandra J, et al. [30]	28	SAA	>18	EPAG/ATG	150 mg
	Lum SH, et al. [31]	48	SAA	>18	EPAG/ATG	150 mg
	Jeong DC, et al. [32]	52	SAA	>18	EPAG/ATG	150 mg
	Tang X, et al. [33]	52	SAA	>18	EPAG/ATG	150 mg
Groake EM, Patel BA, Gutierrez-Rodriguest, et al.: Eltrombopag added to immunosuppression for children with treatment-naïve severe aplastic anemia. (2021)	Groarke EM, et al. [4]	131	SAA	>18	EPAG/ATG/CSA	150 mg
		24	SAA	12-18	EPAG/ATG/CSA	150 mg
		16	SAA	<12	EPAG/ATG/CSA	75 mg/daily (6-11 years) 2.5 mg/kg (2-5 years)
Total	20	1,018	-	-	-	-

An OR of one means there is no relationship between treatment and outcome. An OR greater than one (OR > 1) suggests a positive relationship between treatment and outcome, while an OR less than one (OR < 1) indicates a negative relationship. To summarize data from multiple RCTs and display them on one graph, we used a forest plot. The x-axis represents the OR value, while the y-axis displays the results from individual RCT. Each horizontal line on the graph represents the 95% CI of an individual RCT, with the range extending from the lower to the upper limit of the CI. The CI shows how confident we are in the estimated variation.

The difference between experimental and control groups is analyzed using z-test and p-values. The difference is considered statistically significant if the value of the z-test is between -1.96 and +1.96 (-1.96 < Z-test > +1.96) for p < 0.05 and 95% CI.

Results

We conducted a meta-analysis of three published articles [1,4-5] that reported the efficacy of EPAG for treating SAA when used with standard IST. These articles were published between January and March 2021, and their authorship and title are provided in Table 2. To evaluate the effectiveness of EPAG in combination with other immunosuppressive (IS) drugs for treating SAA, we compared it directly to standard IST. Patients who received the combination of EPAG and standard IST were the experimental group, while those who received standard IST alone were the control group. We compared the hematologic ORR for three to six months and the RR within two years after treatment in both groups.

As shown in Table 2, the selected journals presented data from different RCTs. Therefore, we collected data from a total of 20 RCTs. Patients were randomly assigned to either the experimental or control group in all RCTs. In one RCT, patients in the experimental group were divided into three cohorts based on two criteria: the start date and end date for receiving EPAG. Cohort 1 received EPAG from day 14 to avoid liver toxicity, cohort 2 received EPAG for three months to limit the chance of clonal evolution development, and cohort 3 received EPAG from day one and continued for six months [15]. In another RCT that included children aged two to 18 years, EPAG was administered in different doses recommended for specific age groups [4]. In Table 1, we have provided a list of selected journals, a comprehensive list of trials included in the meta-analysis, and the general characteristics of patients treated with EPAG and IST combination. This includes the total number of study participants in each experimental group, the medical conditions for which patients were treated, the age of patients, the treatment regimen, and the EPAG dose for each group.

A total of 1,018 participants took part in the study across all experimental groups. This included 978 adult patients and 40 children. The adult patients were given EPAG in doses of 150 mg per day in addition to the standard IST treatment. The standard IST consisted of either ATG alone or a combination of ATG and CSA. The children were given a combination of EPAG and ATG/CSA, with doses tailored to their specific age group. Children between two and five years received EPAG in doses of 2.5 mg/kg, while children from six to 11 received 75 mg/day. The EPAG dose of 150 mg/day was assigned to children older than 12 years.

**Figure 2:**
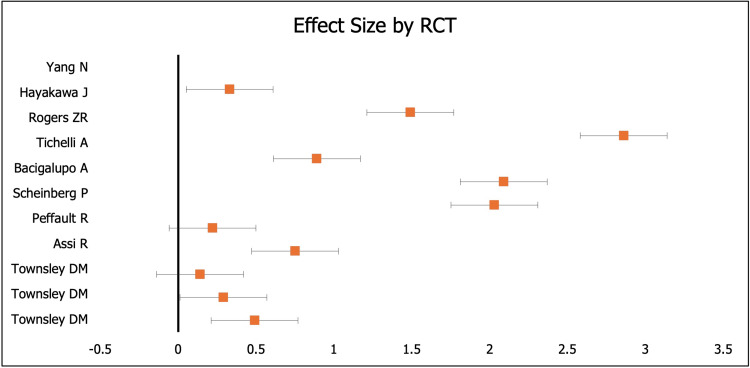
A forest plot showing the distribution of overall response rates in patients on eltrombopag and immunosuppressive therapy or immunosuppressive therapy alone

We reviewed data from 20 RCTs and assessed the overall response and RRs of patients treated for SAA. However, we found data available in only nine RCTs (Table 2 and Table 3). The analysis of data on the ORRs after six months of treatment was conducted for both experimental and control groups. The total number of cases examined was 2,469, with 1,018 patients in the experimental group and 1,451 patients in the control group. The control group received a standard IST regimen that included either ATG alone or a combination of ATG and CSA. In total, there were 1,306 adult patients and 145 children. We performed a chi-squared test of heterogeneity to confirm the consistency of ORR results across the trials included in our meta-analysis. The results of this test (χ^2^ = 2.14, degrees of freedom (DF) = 10, and p > 0.01) showed that ORR results were consistent across the RCTs. The extent of heterogeneity was validated by Cochran’s homogeneity test and I^2^ test. Both tests (Q = 2.83, p < 0.001; I^2^ > 50%) indicated that there was high variability in ORR results across trials. A visual effect size of ORR data in each RCT is presented in a forest plot below (Figure 2).

**Table 3:** Overall response rates in patients on eltrombopag and immunosuppressive therapy or immunosuppressive therapy alone Heterogeneity: χ^2^ = 2.14, DF = 10 (p > 0.01); test for the overall effect: z = 10.270 (p < 0.0001) ORR_E, the overall response rate in the experimental group; ORR_E%, the overall response rate in the experimental group in percentage; ORR_C, the overall response rate in the control group; ORR_C%, the overall response rate in the control group in percentage

Randomized controlled trial	ORR_E	Total	ORR_E%	ORR_C	Total	ORR_C%	Odds ratio (95% CI)
Townsley DM, et al. [15]	24	30	80	61	92	66	0.49 (0.18-1.33)
	27	31	87	61	92	66	0.29 (0.09-0.91)
	29	31	94	61	92	66	0.14 (0.03-0.61)
Assi R, et al. [18]	16	21	76	12	17	71	0.75 (0.18-3.20)
Peffault de Latour R, et al. [19]	73	96	76	41	101	41	0.22 (0.12-0.40)
Scheinberg P, et al. [6]	22	26	85	19	26	73	2.03 (0.51-8.00)
Bacigalupo A. [20]	46	48	96	44	48	92	2.09 (0.36-12.00)
Tichelli A, et al. [8]	31	36	86	28	32	88	0.89 (0.22-3.63)
Rogers ZR, et al. [21]	32	36	89	28	38	74	2.86 (0.81-10.13)
Hayakawa J, et al. [22]	22	26	85	18	26	69	2.44 (0.03-9.45)
Yang N, et al. [23]	33	38	87	31	38	82	1.49 (0.43-5.19)
Total	881	1,018	X̅ = 86	987	1,451	X̅ = 74	0.33 (0.27-0.41)

After confirming the heterogeneity of ORR results, different statistical tests were conducted to validate the data. The OR and 95% CI were calculated for each RCT to determine whether there was an association between the treatment regimen and ORR. The calculated OR indicated a positive correlation between ORR and treatment regimen (OR > 1). However, OR was higher in patients who received IST combined with EPAG compared to those who received IST alone (OR = 0.33 and 95% CI in a range from 0.27 to 0.41). This difference was statistically significant based on the value for the z-test (z = 10.270, p < 0.0001). The hematologic response was significantly improved in SAA patients when EPAG was added to standard IST. The mean ORR was higher in the experimental group (86%) than in the control group (74%). Table 3 presents the data.

**Figure 3:**
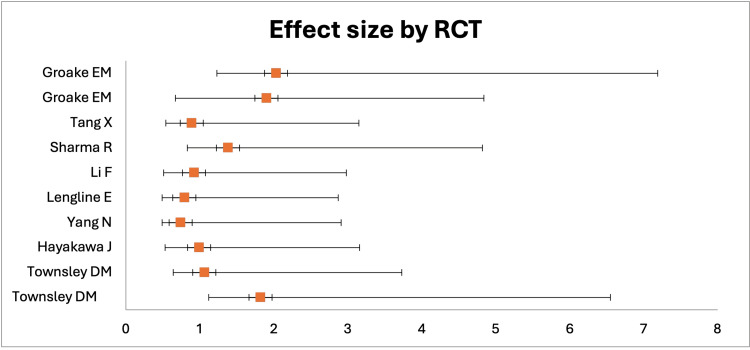
A forest plot showing the distribution of relapse rates in patients on eltrombopag and immunosuppressive therapy or immunosuppressive therapy alone

Data on the rates of SAA relapse were available in only nine RCTs and included a total of 1,179 cases. Out of these patients, 462 were in the experimental group, and 717 were in the control group. The RRs within two years after treatment varied across the RCTs, which was confirmed by the p-value of the chi-squared test (p > 0.01) (χ^2^ = 1.06, DF = 10) and Cochran’s homogeneity test (p < 0.10) (Q = 1.24, p = 0.041). However, the I^2^ test showed low heterogeneity (I^2^<25%). We have presented the visual effect size of RR data for each RCT in a forest plot below (Figure 3).

The study found a positive correlation between RR and treatment regimen as the value of OR was 1.455, and 95% CI was in the range from 1.14 to 1.84, indicating that the use of EPAG could potentially increase RR in SAA patients. However, the z-test (z = 0.021) and the p-value (p = 0.0025) showed that there was no statistically significant difference (p < 0.05) between the RR of the experimental groups treated with EPAG in combination with IST and the control groups treated with IST alone. Despite this, the mean of RR in the group treated with EPAG combined with IST (54 %) was higher than in the group treated with IST alone (39%). To view the data, please refer to Table 4.

**Table 4:** Relapse rates in patients on eltrombopag and immunosuppressive therapy or immunosuppressive therapy alone Heterogeneity: χ^2^ = 1.06, DF = 10 (p < 0.05); test for the overall effect: z = 0.021 (p = 0.0025) RR_E, the relapse rate in the experimental group; RR_E%, the relapse rate in the experimental group in percentage; RR_C, the relapse rate in the control group; RR_C%, the relapse rate in the control group in percentage

Randomized controlled trials	RR_E	Total	RR_E%	RR_C	Total	RR_C%	Odds ratio (95% CI)
Townsley DM, et al. [15]	13	24	54	24	61	39	1.82 (0.70-4.73)
	11	27	41	24	61	39	1.06 (0.42-2.67)
Assi R, et al. [18]	4	16	25	1	12	8	9.00 (0.44-185.37)
Hayakawa J, et al. [22]	28	52	54	27	50	54	0.99 (0.46-2.17)
Yang N, et al. [23]	10	28	36	12	28	43	0.74 (0.25-2.17)
Lengline E, et al. [24]	15	34	44	16	32	50	0.79 (0.30-2.08)
Li F, et al. [26]	26	48	54	27	48	56	0.92 (0.41-2.06)
Sharma R, et al. [27]	18	36	50	16	38	42	1.38 (0.55-3.44)
Tang X, et al. [33]	14	38	37	15	38	39	0.89 (0.35-2.26)
Groarke EM, et al. [4]	54	131	41	77	286	27	1.90 (1.23-2.94)
	12	28	43	17	63	27	2.03 (0.80-5.16)
Total	205	462	X̅ = 54	256	717	X̅ = 39	1.45 (1.14-1.84)

Discussion

The etiology of acquired AA is not yet fully understood, and it may involve different immunopathogenic mechanisms. Patients with AA usually experience symptoms related to bone marrow failure and cytopenias [1-5]. Those with SAA have a significantly reduced number of bone marrow cells, which requires urgent medical attention to prevent severe complications such as opportunistic infections, sepsis, and severe bleeding [1,3]. Currently, there are two main treatments available for SAA: IST and HSCT [7]. While HSCT is the only curative treatment, it is mainly recommended for younger patients with a fully matched HLA family donor or a suitable unrelated donor [4]. The second group of candidates for HSCT is patients who did not respond to the first line of IST. In such cases, bone marrow from unrelated donors is most often used as a source for HSCT [20].

Based on data from post-bone marrow transplant, the chances of long-term survival decrease with the patient’s age. In young children, the long-term survival rate is about 90%, while in adolescents, it is about 80%. However, for patients over 40 years of age, the long-term survival rate drops to 50% [1,4]. On the other hand, for adult SAA patients, IST is the most successful treatment and is recommended as the first line of treatment [20,22-23]. In children, IST is only recommended if there is no suitable HLA-matched family donor. Furthermore, IST can be used as a second-line therapy for patients who experience a relapse of AA after the first cycle of therapy [26-27,30].

Hematologic Response Rates

IS drugs like horse ATG (hATG) and CSA are recommended therapies for acquired AA due to their immunopathogenesis [6-7,22-23,25-26]. It usually takes at least four to 12 weeks of treatment for patients to respond and improve blood counts after initiating therapy [1,3]. Acquired AA can be categorized into SAA and refractory AA (rAA) [7]. Patients with rAA do not respond to the first line of IST, and their severe cytopenias may persist, making them more prone to severe complications and long-term transfusion dependence [16]. In the last decade, the recommended treatment for rAA was the combination of standard IST and other lymphotoxic agents, such as cyclophosphamide, alemtuzumab, and mycophenolate, which present various treatment challenges. However, the current data did not support this treatment regimen due to insignificant improvement in response rates in patients with rAA. The rationale behind this lack of response was that the bone marrow of these patients does not have enough HSCs to produce blood cells [13]. To address this, new clinical trials included different growth factors such as erythropoietin, granulocyte colony-stimulating factor (G-CSF), stem cell factor, and interleukins to restore bone marrow. Unfortunately, none of these growth factors showed promising results in restoring bone marrow [8]. Recently, EPAG has been proposed as a TPO receptor agonist in combination with IST for AA treatment [15-16,18-19,24,27-31]. TPO is a glycoprotein synthesized in the liver that stimulates megakaryocytes (precursors of platelets) and increases platelet production by binding to the c-Mpl receptor expressed on the megakaryocyte membrane. This TPO effect was previously used in the treatment of ITP [9-10]. Several in vitro studies have also demonstrated that the c-Mpl receptor is expressed on the membrane of all bone marrow cells, indicating that TPO can enhance the proliferation and maintenance of HSCs [12].

Recent experiments with TPO knockout mice have shown a significant reduction in all three lineages, not just megakaryopoiesis [13]. Studies in humans have also confirmed the vital role of TPO in the proliferation and maintenance of all HSCs. Mutations in the c-Mpl gene resulted in multi-lineage cytopenias. Genetic analysis of bone marrow cells in patients with AA has revealed that the mutation in the c-Mpl gene was associated with the presentation of disease in some patients [14]. Furthermore, patients with AA have a high serum level of TPO due to the effect of TPO agonists in an increasing dose-dependent study, showing that the elevated endogenous TPO serum levels can be overcome by using a high-dose of an oral TPO agonist such as EPAG [16]. Therefore, EPAG seems to be an ideal therapeutic agent for AA because of its beneficial effects on both the expansion of HSCs and hematopoietic progenitor cells. Consequently, this drug may enlarge the reserve of HSCs, which are markedly reduced in patients with AA. Nonetheless, there is strong clinical and theoretical evidence that EPAG can help restore hematopoiesis in SAA by stimulating HSCs [1,13]. Among other TPO receptor agonists, EPAG has been found to be the most effective in treating AA [31]. It is a synthetic, non-peptide TPO mimetic that closely resembles TPO in terms of its binding pathways to the c-Mpl receptor, though at different domains. EPAG selectively binds to the trans-membrane and juxta-membrane domains of the TPO receptor, not competing for binding with the endogenous TPO [10]. The published data confirm the effectiveness of EPAG in restoring hematopoiesis, making it a recommended first-line treatment for SAA and especially for treatment-naïve patients with rAA, in addition to standard IST [1,4-5,16,19]. A single-drug clinical efficacy test was conducted on 43 patients with rSAA who had previously shown no response to at least one course of IST. In this non-randomized phase II dose-escalating study, patients were treated with an initial dose of 50 mg of EPAG, which was increased by 25 mg every two weeks to a maximum dose of 150 mg over six months. After three to four months of treatment, 40% of patients achieved an ORR (complete response plus partial response) [16]. Recently, another study was conducted to evaluate the effectiveness of EPAG in combination with standard IST in patients with moderate aplastic anemia (MAA) and SAA. The study found that EPAG was more beneficial in patients with MAA than in SAA patients. The initial dose of EPAG was 50 mg and was increased to 300 mg. After 14-20 weeks of treatment, the ORR was 50%, but most of these patients experienced a relapse and required re-induction of their treatment. The more favorable response in patients with MAA can be attributed to their higher HSC reserve in their bone marrows compared to SAA patients [34]. Since the results were not satisfactory, subsequent studies were conducted to test the efficacy of EPAG given in combination with standard IST (ATG and CSA) for six months. The initial dose of EPAG was 150 mg, selected from previous results from the dose-escalation trials. Patients were assigned to three treatment groups. The first group (cohort 1) started to receive EPAG from day 14 to help prevent liver toxicity as a possible side effect of long-term therapy with ATG/CSA. The second group (cohort 2) was treated for three months to avoid the potential of clonal evolution, a well-known long-term side effect of IST. The complete response rate in this group significantly decreased from 33% to 26%, likely due to the shorter treatment period with EPAG. The third group (cohort 3) received a combination of EPAG and IST from the first day of treatment up to six months. This group achieved the highest overall hematologic response rate of 95% and a complete response rate of 58% after six months of treatment. These results demonstrate that combined treatment with EPAG and IST is superior to historical data, which showed an overall hematologic response of 66% and a complete response of 10% [15]. However, the benefit of adding EPAG to IST was not confirmed in subsequent clinical trials. In these trials, response rates were assessed in two experimental groups. The first group received only IST, while the second group received the combination of EPAG, IST, and growth factor. The difference in response rates between these groups was not statistically significant (71% vs. 76%, respectively). It is possible that the different trial designs influenced the outcomes and made the results not representative. In the subsequent trial, EPAG was initiated later (from day 14) and continued to be administered for longer than six months. CSA was given for a shorter duration of no longer than six months. Additionally, the response rate was assessed for a longer follow-up period (21 months vs. six months). Lastly, there were differences in the definition of hematologic response and the time points of response assessed [8]. The European Group for Blood and Bone Marrow Transplantation (EBMT) conducted a study to compare the effectiveness of standard IST with and without EPAG. This was a prospective, randomized, multicenter phase III trial in which EPAG was given from day 14 for three to six months. The results of the study demonstrated a substantial statistical discrepancy in ORRs between the two groups. The group that received EPAG in addition to standard IST had a response rate of 76%, compared to 50% in the group that received standard IST alone over a six-month period. The study highlights that the combination of EPAG and standard IST is more effective than treatment with IST alone [19]. In subsequent studies, it was found that adding EPAG to the current standard IST has advantages in terms of response time. Patients who received EPAG had a more rapid reconstitution of hematopoiesis, resulting in fewer complications related to severe cytopenias. As a consequence, these patients required fewer blood transfusions [24]. Another study confirmed that the combined therapy of IS drugs was more effective than single-drug therapy. The synergy between those drugs was easily achieved, but the studies did not explore whether the beneficial effects of adding EPAG to the standard IST were related to the synergistic effects of EPAG with other drugs [18]. It seems that treatment with the combination of EPAG and IST is superior to treatment with IST alone, but further investigations are needed to understand the potential interactions between these drugs. The studies also did not define the optimal dose of EPAG or the duration of hematologic response to this drug [1,4-5]. In a study by the National Institutes of Health (NIH), rSAA patients who showed a trilineage response to EPAG had the potential for sustained stable blood counts even after stopping treatment. This may be attributed to the fact that patients who had a favorable response to EPAG were able to regenerate a crucial number of HSCs and continue producing bone marrow cells without the need for treatment [16]. In many studies, a dose of 150 mg given for 24 weeks was found to be the most effective, primarily for patients who did not respond to IST after 12 weeks of treatment [18-19]. However, it is important to confirm if a reduced dose response of EPAG can achieve the same results in future clinical studies. The extended duration of follow-up data from the NIH trial, with a median follow-up of two years in SAA patients who were treated with EPAG, did not confirm the previously published data. The ORRs of patients who were treated with a combination of EPAG and IST were similar to those in patients who were treated with standard alone. It remains unknown if the prolonged hematologic response rate depends on EPAG’s benefit in stimulating HSCs or if it is a response to IST [32].

Due to the lack of consistency in published data about EPAG efficacy, we conducted a meta-analysis to compare the ORRs in SAA patients treated with standard IST versus a combination of EPAG and IST. The analysis included data from three medical journals. Each study grouped patients into experimental and control groups based on their assigned treatment. The dataset included ORR from a total of 2,469 patients. Among them, 1,018 patients were in the experimental group, and 1,451 patients were in the control group. The majority of patients (N = 1,306) were adults, while 145 were children aged 12 to 18 years. The significant number of patients in both groups provided the statistical power for the comparison. The experimental group showed a higher ORR compared to the control group (86% vs. 74% retrospectively). Statistical tests (chi-squared test and z-test) confirmed that the difference between the two groups was statistically significant (χ^2^ = 2.14 and z-test = 10.27, p < 0.0001). This suggests that EPAG, in combination with IST, is a more effective and superior treatment for SAA than IST given alone. Another category with treatment challenges is the group of children with SAA, especially as they often lack a matched-sibling donor. Initial IST is the primary treatment for this group [26]. While studies have shown high success rates with IST, there is also a high percentage of relapse and clonal malignant myeloid transformations (CMMT) associated with it [18]. Recently, researchers investigated the potential benefits of adding EPAG to standard IST for SAA treatment in children. Surprisingly, the efficacy of EPAG in children was found to be lower than in adult patients, with response rates of less than 80%. It is unclear why these results differ, especially considering that children had better response rates to standard initial IST and tolerated more intensive IST regimens well. In addition, this study found differences in response rates between younger children (≤12 years of age) and adolescents, attributing this to biological differences. In conclusion, the data from this non-randomized trial did not confirm the benefits of adding EPAG to standard IST for treating SAA in children [4]. It is necessary to conduct further investigation to determine whether EPAG can have a positive impact on the outcomes of children with SAA. In our meta-analysis, we compared the ORR between the adult and children experimental groups. The findings indicated that the response to combine therapy with EPAG and IST was stronger in adults than in children. The ORR was found to be higher in adult patients (88%) compared to children (73%), consistent with prior studies. In several studies, both the treatment effectiveness and cost-effectiveness of the treatment with EPAG were evaluated. The costs of using EPAG at doses of 150 mg/day for a six-month treatment period were calculated. Depending on the treatment response, some patients achieved a complete response and continued with low-dose CSA for up to one year. The second group consisted of non-responders who continued to receive EPAG for an additional six months. A comparison between these groups revealed that treatment with EPAG for longer than six months increased the costs. According to the Therapeutic Advances in Hematology USA, the use of EPAG increased the costs by about $50 million over three years. Knowing the synergistic effects of EPAG with other drugs, the researchers suggested using EPAG in combination with IST. Combined therapy significantly reduced the costs of SAA treatment since the dose of EPAG was lower when combined with IST [18].

Relapse Rates

In previous studies, SAA patients treated with IST as a first line of therapy demonstrated elevated RRs following treatment cessation. Currently, CSA is the recommended drug for the treatment of SAA relapse. However, long-term therapy with CSA is compromised with numerous complications and disadvantages. First, the hematologic response to CSA is very slow, and it usually takes years for patients to reach a complete or partial response. Furthermore, extended treatment of CSA is associated with a greater risk of experiencing side effects. Additionally, CSA can lead to patients becoming dependent on their blood count. Lastly, this drug may cause significant lymphocyte depletion and require switching CSA to other drugs (ATG or alemtuzumab) that usually have more liver and kidney toxicity [24].

Recently published data confirmed that EPAG is effective in the treatment of AA, and additionally, it may prevent relapse of SAA. These results indicate that EPAG has a safety profile and, therefore, can be administered safely to SAA patients for a long time [1]. The other studies compared the RRs of patients who received IST alone to those receiving a combination of EPAG and IST. Among those, the NIH study showed that the treatment-naïve SAA patients had a higher RR compared to the group of patients with IST alone(30-40% vs. 54% retrospectively). In this group of patients, the treatment with EPAG started from day 14 up to a six-month period [7]. Moreover, the results indicated that the treatment with low doses of CSA can decrease RR by 15%. Based on these results, it was confirmed that there was no difference in RRs of patients who received EPAG combined with IST compared to those patients who received IST alone. The follow-up period of two years post-treatment was used to calculate RRs [15]. Our results were consistent with these data. When comparing the two groups, we found that patients who received EPAG combined with IST had higher RRs than those who received IST alone (54% vs. 39%, respectively). However, the statistical tests (χ^2^ = 1.06, DF = 10; z-test = 0.021, p = 0.0025) confirmed that this difference was not statistically significant(p < 0.05). Based on our meta-analysis, EPAG shows limited effectiveness in lowering RRs in SAA patients, even when used as a long-term treatment. Most importantly, the NIH study discovered that the peak in relapse was observed at six months and two years after discontinuing EPAG or CSA treatment. This suggests that RR is inversely correlated to EPAG treatment duration. The study compared RRs at six months and two years post-treatment periods. The results demonstrated that the RR was higher in the group of patients who received EPAG for six months compared to those who received EPAG for longer than two years. Moreover, patients who received EPAG for longer than two years remained stable and improved their blood counts after treatment discontinuation [32]. Further research confirmed that a gradual stepwise approach to discontinuing EPAG therapy resulted in higher reductions in RRs compared to abrupt discontinuation. Therefore, it is recommended that the EPAG dose should be tapered off gradually like CSA waning. The use of EPAG for extended periods (⩾ two years) and a gradual decrease in dosage may have a positive impact on preventing relapse in SAA patients [4,18].

Adverse Reactions

The data from various clinical trials provided information about possible adverse events and reactions during EPAG treatment [4-5,28,32]. The studies confirmed that EPAG is better tolerated in SAA patients, even at higher doses than prescribed for ITP treatment [16,24]. Safety and efficacy data were collected at six months or one year after treatment with EPAG. Adverse reactions, such as skin, gastrointestinal, and hepatic impairment, were reported by most patients during treatment, with the majority experiencing mild to moderate symptoms [1]. However, recent studies with extended follow-up data discovered that EPAG has the potential to increase the development of bone marrow clones and progress to the clonal and malignant myeloid transformation (CMMT) of bone marrow cells. Bone marrows were collected from SAA patients who underwent a two-year treatment, either with a combination of EPAG and IST or IST alone. The examinations indicated an increase in the number of clones associated with paroxysmal nocturnal hemoglobinuria (PNH), myelodysplastic syndrome (MDS), and acute myeloid leukemia (AML). The incidence rate of CMMT was in a range from 5% to 15% over five to 12 years of disease follow-up. Nonetheless, the incidence rates of clonal evolution did not differ significantly between patients who received EPAG and IST and those who received IST alone [18]. Another study showed that patients with somatic mutations in myeloid cancer genes have a higher risk of developing CMMT than those with no mutations. The prevalence rate in these patients was 45%. Likewise, patients with rSAA who received EPAG for over two years had a higher likelihood of developing different aberrations in their karyotype. The most frequent aberrations were chromosome 7 aberrations and complex MDS/AML clone transformations. They occurred in 18% of patients. Interestingly, these aberrations (87%) tended to emerge very early during treatment, usually within the first six months [32]. Additionally, the genetic studies demonstrated that the loss of the sterile alpha-domain 9 and sterile alpha-domain 9-like genes were triggers for initiating CMM in mice treated with EPAG. Both genes are located on chromosome 7 [11-12]. According to results from another study, EPAG has the potential to stimulate dormant clones with an aberrant karyotype [13]. Recently, exome sequencing studies discovered that SAA patients may have somatic mutations similar to myeloid malignancies. Hence, the findings imply that immune-related mechanisms may contribute to genetic mutations in SAA patients. The most common mutations were PIGA mutation and uniparental disomy for the short arm of chromosome six [14]. The studies in children with SAA showed that the children treated with a combination of EPAG and IST had the same rate of development CMMT compared to the controls who received only IST. However, the rate of CMMT in children treated with EPAG alone was higher than in adults, but this difference did not reach statistical significance [18]. Due to a lack of reporting CMMT data in selected publications, we have not assessed the clonal evaluation rate in our review. Despite multiple studies confirming the potential for bone marrow clone development in SAA patients, the impact of EPAG therapy on CMMT remains uncertain. Currently, there are two possible theories. According to the first theory, EPAG may cause HSCs to proliferate excessively, leading to a higher risk of somatic mutations in immature bone marrow cells. The second theory suggests that somatic mutations are caused by a selection process when HSCs proliferate excessively [4-5]. Various experiments have indicated that EPAG could play a protective role and induce rapid cell death in pre-leukemic and leukemic cells by decreasing reactive oxygen levels. Further data demonstrated that EPAG has the ability to impede the proliferation of leukemia cells by decreasing intracellular iron and stimulating cell differentiation. Until more evidence is obtained, it is currently recommended that SAA patients receiving EPAG therapy should have regular follow-ups after treatment. In most cases, the follow-up assessment requires bone marrow examination and genetic studies, including cytogenetics and molecular genetics. These examinations aim to detect CMMT early and prevent the progression of MDS/AML clones in SAA patients [1,4-5].

Strengths and Limitations

The review results strongly confirm that EPAG significantly improves hematologic response in SAA patients if combined with standard IST. Therefore, it is a superior treatment option compared to standard IST alone. These results have a significant impact on data validation as they included information on the effectiveness of EPAG in large populations. Additionally, variations in study design, inclusion/exclusion criteria, and study parameters among RCTs may impact the study results.

Furthermore, to select the journals, we only searched the PubMed online database and included journals that met our selection criteria: (1) journals published within the last year, (2) studies conducted on human participants, (3) available in the free full-text format, and (4) written in English. These criteria significantly limited our search to only three journals. In this review, we did not analyze the long-term effects of EPAG therapy on bone marrow clonal evolution since these data were presented only in one journal. Future studies need to include additional data and re-validate the benefits and side effects of using EPAG in combination with standard IS drugs for treating SAA.

## Conclusions

In conclusion, the EPAG combined with standard IST is a more effective treatment for SAA patients than IST alone. Our meta-analysis confirmed that 86% of SAA patients improved blood counts after six months of treatment with EPAG and standard IST. This percentage was significantly higher than in patients treated with standard IST alone (76%). The overall hematologic response rate improvement was achieved due to the EPAG and IS drug's synergistic effects. When used as a single drug, EPAG was insufficient to improve the ORR in SAA patients, and only 40-50% of these patients had a partial response. Aside from the synergistic effects, the major benefit of adding EPAG to standard IST is a rapid response in SAA patients. This is critical for patients with very low blood counts who are susceptible to severe complications. The combined therapy of EPAG and standard IST has mainly been proven to benefit adult SAA patients or patients who did not respond to the first line of IST. The promising effects of combined therapy with EPAG and standard IST have also been tested in children. Around 70% of children with SAA treated with EPAG and standard IST showed improvement in their blood counts after six months of treatment. EPAG provided the best outcomes in adult SAA patients when given as a short-term treatment at a dose of 150 mg daily. Our meta-analysis concluded that 54% of patients treated with EPAG and standard IST had SAA relapse after two years of treatment. The RRs were slightly lower in the group of patients who were treated with standard IST alone (39%). Patients who received a low dose of EPAG for longer than two years continued to have stable blood counts after treatment discontinuation. There is evidence that EPAG can increase the proliferation of bone marrow progenitor cells and regain the reserves of HSCs in bone marrow. Nevertheless, future investigations should determine the most effective EPAG dose for treating SAA in adults and children while minimizing long-term side effects.

## References

[1] Drexler B, Passweg J (2021). Current evidence and the emerging role of eltrombopag in severe aplastic anemia. Ther Adv Hematol.

[2] Urbanowicz I, Nahaczewska W, Celuch B (2021). Narrative review of aplastic anemia-the importance of supportive treatment. Ann Palliat Med.

[3] Young NS (2018). Aplastic anemia. N Engl J Med.

[4] Groarke EM, Patel BA, Gutierrez-Rodrigues F (2021). Eltrombopag added to immunosuppression for children with treatment-naïve severe aplastic anaemia. Br J Haematol.

[5] Zhang J, Wu Y, Liu J, Han S, Chen L, Wang H, Peng Y (2021). A systematic review and meta-analysis of the safety and efficacy of anti-thymocyte globulin combined with eltrombopag in the treatment of severe aplastic anemia. Ann Palliat Med.

[6] Scheinberg P, Nunez O, Weinstein B, Scheinberg P, Biancotto A, Wu CO, Young NS (2011). Horse versus rabbit antithymocyte globulin in acquired aplastic anemia. N Engl J Med.

[7] Young NS, Calado RT, Scheinberg P (2006). Current concepts in the pathophysiology and treatment of aplastic anemia. Blood.

[8] Tichelli A, de Latour RP, Passweg J (2020). Long-term outcome of a randomized controlled study in patients with newly diagnosed severe aplastic anemia treated with antithymocyte globulin and cyclosporine, with or without granulocyte colony-stimulating factor: a Severe Aplastic Anemia Working Party Trial from the European Group of Blood and Marrow Transplantation. Haematologica.

[9] Bussel JB, Cheng G, Saleh MN (2007). Eltrombopag for the treatment of chronic idiopathic thrombocytopenic purpura. N Engl J Med.

[10] Garnock-Jones KP, Keam SJ (2009). Eltrombopag. Drugs.

[11] Zeigler FC, de Sauvage F, Widmer HR (1994). In vitro megakaryocytopoietic and thrombopoietic activity of c-mpl ligand (TPO) on purified murine hematopoietic stem cells. Blood.

[12] Ku H, Yonemura Y, Kaushansky K (1996). Thrombopoietin, the ligand for the Mpl receptor, synergizes with steel factor and other early acting cytokines in supporting proliferation of primitive hematopoietic progenitors of mice. Blood.

[13] Alexander WS, Roberts AW, Nicola NA (1996). Deficiencies in progenitor cells of multiple hematopoietic lineages and defective megakaryocytopoiesis in mice lacking the thrombopoietic receptor c-Mpl. Blood.

[14] Ihara K, Ishii E, Eguchi M, Takada H, Suminoe A, Good RA, Hara T (1999). Identification of mutations in the c-mpl gene in congenital amegakaryocytic thrombocytopenia. Proc Natl Acad Sci U S A.

[15] Townsley DM, Scheinberg P, Winkler T (2017). Eltrombopag added to standard immunosuppression for aplastic anemia. N Engl J Med.

[16] Olnes MJ, Scheinberg P, Calvo KR (2012). Eltrombopag and improved hematopoiesis in refractory aplastic anemia. N Engl J Med.

[17] Page MJ, McKenzie JE, Bossuyt PM (2021). The PRISMA 2020 statement: an updated guideline for reporting systematic reviews. PLoS Med.

[18] Assi R, Garcia-Manero G, Ravandi F (2018). Addition of eltrombopag to immunosuppressive therapy in patients with newly diagnosed aplastic anemia. Cancer.

[19] Peffault de Latour R, Kulasekararaj A, Iacobelli S (2022). Eltrombopag added to immunosuppression in severe aplastic anemia. N Engl J Med.

[20] Bacigalupo A (2019). Antithymocyte globulin and cyclosporin: standard of care also for older patients with aplastic anemia. Haematologica.

[21] Rogers ZR, Nakano TA, Olson TS (2019). Immunosuppressive therapy for pediatric aplastic anemia: a North American Pediatric Aplastic Anemia Consortium study. Haematologica.

[22] Hayakawa J, Kanda J, Akahoshi Y (2017). Meta-analysis of treatment with rabbit and horse antithymocyte globulin for aplastic anemia. Int J Hematol.

[23] Yang N, Chen J, Zhang H (2017). Horse versus rabbit antithymocyte globulin in immunosuppressive therapy of treatment-naïve aplastic anemia: a systematic review and meta-analysis. Ann Hematol.

[24] Lengline E, Drenou B, Peterlin P (2018). Nationwide survey on the use of eltrombopag in patients with severe aplastic anemia: a report on behalf of the French Reference Center for Aplastic Anemia. Haematologica.

[25] Sasaki N, Shimura K, Yoshida M (2019). Immunosuppressive therapy with rabbit antithymocyte globulin therapy for acquired aplastic anemia: a multi-institutional retrospective study in Japanese adult patients. Int J Hematol.

[26] Li F, He W, Shi W, Xie X (2020). Efficacy of rabbit antithymocyte globulin as a first-line therapy in children with aplastic anemia. J Pediatr Hematol Oncol.

[27] Sharma R, Chandra J, Sharma S, Pemde H, Singh V (2012). Antithymocyte globulin and cyclosporine in children with aplastic anemia: a developing country experience. J Pediatr Hematol Oncol.

[28] Bevans MF, Shalabi RA (2004). Management of patients receiving antithymocyte globulin for aplastic anemia and myelodysplastic syndrome. Clin J Oncol Nurs.

[29] Frickhofen N, Rosenfeld SJ (2000). Immunosuppressive treatment of aplastic anemia with antithymocyte globulin and cyclosporine. Semin Hematol.

[30] Chandra J, Naithani R, Ravi R (2008). Antithymocyte globulin and cyclosporin in children with acquired aplastic anemia. Indian J Pediatr.

[31] Lum SH, Grainger JD (2016). Eltrombopag for the treatment of aplastic anemia: current perspectives. Drug Des Devel Ther.

[32] Jeong DC, Chung NG, Cho B (2014). Long-term outcome after immunosuppressive therapy with horse or rabbit antithymocyte globulin and cyclosporine for severe aplastic anemia in children. Haematologica.

[33] Tang X, Liu F, Li L (2012). Antithymocyte globulin/antilymphocyte globulin plus kidney-nourishing Chinese medicinal: effect on severe aplastic anemia. J Tradit Chin Med.

[34] Fan X, Desmond R, Winkler T (2020). Eltrombopag for patients with moderate aplastic anemia or uni-lineage cytopenias. Blood Adv.

